# Lymphocyte Count and Body Mass Index as Biomarkers of Early Treatment Response in a Multiple Sclerosis Dimethyl Fumarate-Treated Cohort

**DOI:** 10.3389/fimmu.2019.01343

**Published:** 2019-06-14

**Authors:** Alessia Manni, Antonio Iaffaldano, Giuseppe Lucisano, Mariangela D'Onghia, Domenico Maria Mezzapesa, Vincenzo Felica, Pietro Iaffaldano, Maria Trojano, Damiano Paolicelli

**Affiliations:** ^1^Department of Basic Medical Sciences, Neurosciences and Sense Organs, University of Bari “Aldo Moro”, Bari, Italy; ^2^Center for Outcomes Research and Clinical Epidemiology, Pescara, Italy; ^3^Operative Unit of Neurology, “Dimiccoli” General Hospital, Barletta, Italy

**Keywords:** dimethyl fumarate, lymphocyte count, body mass index, biomarkers, treatment response

## Abstract

**Introduction:** In relapsing Multiple Sclerosis (RMS) patients treated with disease modifying drugs (DMDs), few data are available regarding the biomarkers of treatment response. We aimed to assess the predictive value of lymphocyte count (LC) and Body Mass Index (BMI) for treatment response in a real life setting of dimethyl fumarate (DMF) treated patients.

**Materials and Methods:** We included in our observational analysis 338 patients who were prescribed DMF in an Italian MS Center. We collected clinical and demographic data at the beginning of DMF (T0), and assessed White Blood Cells (WBC) and LC at T0 and at 3 (T3), 6 (T6), 9 (T9), and 12 (T12) months. Gadolinium enhancing (Gd+), new T2 lesions and relapses within the first year of treatment (T12) were recorded in order to evaluate clinical activity at 12 months. Analysis of correlation was performed to correlate WBC, LC and BMI with clinical and radiological responses. We evaluated whether BMI or LC can predict treatment response by using multivariate logistic regression models at each follow-up.

**Results:** Our cohort was followed up for a mean period of 19.8 ± 6.8 months. The mean BMI at baseline was 24.19 ± 4.48. The multivariate models gave as predictive factors for Gd+ lesions at T12, LC at T3 (OR = 1.003, 95% CI = 1.00-1.07; *p* = 0.046) and baseline BMI (OR = 0.71, 95% CI = 0.52–0.98; *p* = 0.037). Predictive factors for new T2 lesions at T12 were LC at T3 (OR = 1.01 95%CI = 1.00–1.95; *p* = 0.005) and baseline BMI (OR = 0.99, 95% CI = 0.98–1.00; *p* = 0.026).

**Conclusions:** In our real life-experience, BMI and LC may be early biomarkers to predict treatment response during DMF.

## Introduction

Multiple Sclerosis (MS) is a chronic immune-mediated disease of the central nervous system mainly affecting young adults (1). The development of disease modifying drugs (DMDs) has made an important contribution to MS treatment, allowing a reduction in the frequency of relapses and delaying disease progression. In recent decades there has been a constant increase in the availability of DMDs with different mechanisms of action, routes of administration and safety profiles. Interferon-β (IFN-β) and glatiramer acetate (GA) are well-established MS therapies which have well-characterized safety profiles and have shown a reduction of relapse rate by approximately one third. However, these DMDs are administered through weekly or daily injections and patients may experience breakthrough disease activity and tolerability may be suboptimal (2, 3). These drawbacks have prompted the search for drugs that could reduce the burden of medication administration and be somehow more efficacious. All newer DMDs have the aim of regulating or suppressing the immune system through specific pathways, and in many cases this has resulted in foreseeable adverse effects (lymphopenia).

Dimethyl fumarate (DMF) is a DMD that was approved for the treatment of relapsing–remitting MS (RRMS) in 2013 in the USA and in 2014 in the European Union. Two Phase III studies and their ongoing long-term extensions have proved that DMF reduces Annualized Relapse Rate (ARR) by ~50% and also reduces the number of new or enlarging T2 hyperintense lesions, new T1 hypointense lesions, and gadolinium-enhancing (Gd+) lesions (4–6). Although its exact mechanism of action has not been fully explained, DMF has proven capable of reducing white blood cell (WBC) counts and absolute lymphocyte counts (LC) by ~30% from the baseline within the first year of treatment (7). Of the patients treated for at least 6 months, 2.2% experienced LC <500 mm^3^ [grade 3 lymphopenia, according to Common Terminology Criteria for Adverse Events –CTCAE- vs. 4.0 (8)] persisting for at least 6 months (5). However, the clinical implications of DMF-induced leukopenia and lymphopenia on treatment response are not yet fully understood. Nevertheless, the need for a careful surveillance is strong given the cases of progressive multifocal leukoencephalopathy (PML) occurring in DMF treated patients with sustained lymphopenia (grade 3 lymphopenia for >6 months) (9).

In addition to acting via the immunomodulation of various cells, DMF also appears to act through neuroprotection since it induces the nuclear factor erythroid 2–related factor 2 (Nrf2) pathway (10). It has been demonstrated that Nrf2 activity is lessened in diabetes and that body weight could modify its activity (11). In recent years, several studies have investigated the role of the Body Mass Index (BMI) in the pathogenesis of autoimmune diseases, such as MS. It has been shown that obesity may worsen the disease course in several autoimmune diseases (12) and studies have reported a positive association between BMI and disability among MS patients (13). Although this evidence supports the role of obesity as a risk factor for MS and its progression, the potential effects of BMI on treatment response have not yet been fully understood. Our work attempted to assess the predictive value of LC and BMI for treatment response in a real-life cohort of DMF-treated relapsing Multiple Sclerosis (RMS) patients. For this purpose, we evaluated the temporal profile of WBC and LC during the first year of treatment and also correlated clinical and Magnetic Resonance Imaging (MRI) (T1 Gd+ lesions, new or enlarging T2 lesions) variables with BMI and with WBC and LC variation in order to identify potential biomarkers of treatment response.

## Methods

### Study Design, Study Population, and Follow-Up

**Figure 1:**
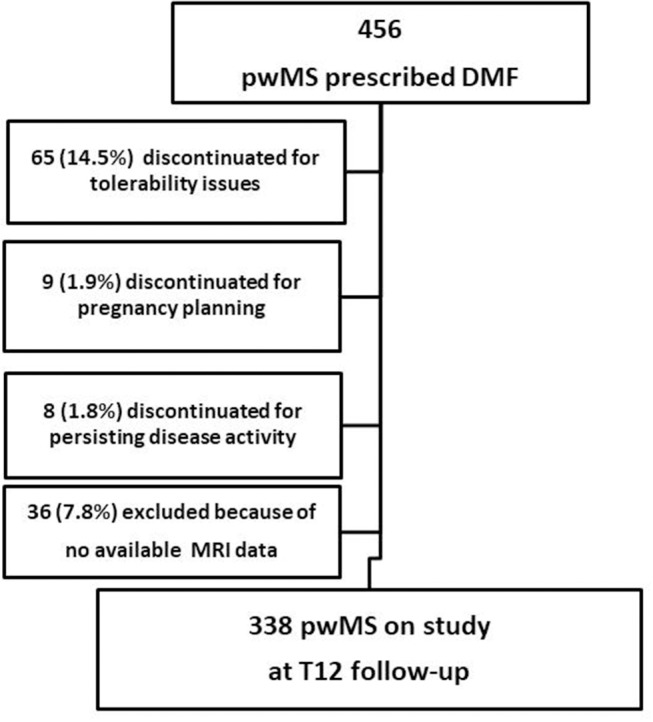
Flow diagram of the study. pwMS, people with Multiple Sclerosis; DMF, dimethyl fumarate; MRI, Magnetic Resonance Imaging; T12, 1 year follow-up.

This is an observational, retrospectively acquired cohort study, approved by the Local Ethics Committee. At the beginning of DMF all patients provided their written informed consent to authorize the use of their clinical and MRI data. A total number of 456 patients started DMF-treatment at the Center for Multiple Sclerosis of the University of Bari between 2014 and 2016; we studied a cohort of 338 patients with RMS according to McDonald and Polman criteria (14, 15), who had reached at least 1-year follow up (Figure 1). Three patients (0.5%) had an unscheduled MRI before the sixth month of treatment (mean time of MRI acquisition = 2.8 ± 0.2 months), showing, according to their treating physician, a high MRI disease activity compared with the baseline MRI; therefore they interrupted the treatment before reaching the sixth month follow up, underwent a high-dose intravenous glucocorticoids, and were then switched to second line DMDs. Five patients (1.1%) experienced a disabling relapse within the third month of treatment and were therefore treated with high dose steroids. Three of these patients were also treated with plasma exchange. All five interrupted DMF treatment. These eight patients were not included in the study cohort.

At the beginning of the treatment (baseline), the following demographic, clinical and MRI data were collected from all patients using the electronic iMed database: age and gender, weight, height, BMI, disease duration, number of relapses, previous DMDs, presence/absence of new/enlarging T2 lesions, and presence/absence of Gd+ lesions. To avoid the slight weight gain (16), possibly caused by a steroid cycle, patients weight and BMI were recorded at least 30 days after the last day of steroids intake. Patients were assessed for neurological disability using the Expanded Disability Status Scale (EDSS) (17) at baseline and at 3-month intervals, and an additional neurological assessment was performed in the event of a relapse. Relapses were defined as episodes of neurological symptoms occurring at least 30 days after the onset of any previous episode, lasting at least 24 h, not attributable to any other causes, occurring in the absence of an infection or fever, and accompanied either by new clinical signs, i.e., changes in the neurological examination, or by an increased EDSS score (1). Peripheral blood (PB) samples were collected in order to monitor WBC and LC at baseline (T0), and then at 3-month intervals. To avoid any possible confounders due to the effect of steroid treatment in the case of relapses/MRI activity, PB samples were collected at least 30 days after the last day of corticosteroids intake. WBC count and LC were evaluated using the quality reference of the same laboratory at the Center for Multiple Sclerosis of the University of Bari.

MRI scans were performed on a 1.5 T Achieva scanner (Philips Healthcare, Best, Netherlands) and 8-channel head coil. Dual-echo sequences (to obtain T2 weighted images; TR 2,800 ms, TE 21/120 ms, flip angle: 90°) and fluid attenuated inversion recovery (FLAIR; TR/TE/TI, 8,900/190/2,500 ms, flip angle: 90°) were acquired in axial orientation (50 slices, 3-mm thick, 1-mm gap, FOV 256 x 256, matrix 400 x 400). Before and after gadolinium administration, T1 weighted images (TR /TE 1,000/12.5 ms, flip angle: 69°) were acquired in axial orientation (22 slices, 5-mm thick, 1-mm gap, FOV 230 ×230, matrix 320 ×320). Spinal cord MRI protocol included T2-weighted (TR/TE 3,500/120 ms) and T1-weighted (before and after gadolinium administration; TR/TE 400/7.5 ms), acquired in sagittal orientation (13 slices, 3.5-mm thick, 0.35-mm gap, FOV 522 ×522, matrix 1,024 ×1,024). Baseline MRI scans were performed no more than 6 months before the start of DMF treatment (mean time of MRI acquisition = 3.8 ± 1.4 months), and then at 6-month intervals (±30 days). MRI outcomes were defined as follows: presence/absence of new/enlarging T2 lesions; presence/absence of Gd+ lesions. Trained neuro-radiologists performed the lesion count by visual analysis of two successive MRI images.

### Clinical Variables

The quantitative variation in WBC and LC, considered both in terms of absolute value and of the “delta” (Δ = the difference between the absolute values of WBC and LC between two consecutive time-points), were analyzed. The baseline (T0) was compared with four different periods of treatment: T3 (3rd month ± 30 days), T6 (6th month ± 30 days), T9 (9th month ± 30 days), and T12 (12th month ± 30 days). Moreover, in order to investigate the impact of the DMF-induced lymphopenia (DIL) on the risk of clinical and MRI activity, the median of the absolute LC was calculated at each observation time, and patients were divided into two groups: those with a high DIL (DIHL, above the median) and those with a low DIL (DILL, below the median). We used this statistical (not clinical) classification to divide the study population into two equal-sized groups (50% of the LC distribution for each group).

### Outcomes

- Presence/absence of new T2 lesions at the T12 MRI follow-up;
- Presence/absence of Gd+ lesions at the T12 MRI follow-up;
- Presence/absence of relapses within the first year of DMF treatment;
- Variation in WBC and LC over time.

### Statistical Analysis

Baseline characteristics were reported for continuous variables as mean ± standard deviation (SD) and for categorical variables as percentages. Characteristics were compared in subjects with and without an event (presence/absence of Gd+ lesions, presence/absence of new T2 lesions, presence/absence of relapses) using the *T*-test for continuous variables and the chi-squared test for categorical variables. For the comparison over time, we used the Wilcoxon matched pair test, and analysis of Pearson correlation was performed to correlate WBC and BMI.

We used multivariate analysis testing the following covariates: sex, age at the start of DMF, presence/absence of T2 lesions at the baseline MRI, presence/absence of Gd+ lesions at the baseline MRI, presence/absence of relapses in the year before starting DMF, previous DMD exposure (naïve patients vs. patients treated with a first-line DMD vs. those treated with a second-line DMD), variation in WBC from T0 to T3, LC at T3, baseline BMI, DIHL at T3 and at T6. Multivariate logistic regression models always considered as covariates the baseline clinical and demographic characteristics of the cohort, and those covariates significant at the univariate analysis, and were used to evaluate whether BMI or LC can predict the outcome. We used a multivariate logistic regression approach for each follow-up because there were no exact dates for follow-up evaluation. Results were expressed as odds ratio (OR) and 95% confidence intervals (95% CI). A 2-sided p <0.05 was considered significant. All analyses were performed using the SPSS version 19.0 (SPSS Inc., Chicago, Illinois).

## Results

The mean ± standard deviation (SD) follow-up of our cohort was 19.8 ± 6.8 months. The baseline demographic and clinical characteristics of the study population are shown in Table 1. Our cohort consisted of 197 (58.3%) female patients and 141 (41.8%) male patients. The mean age at DMF initiation was 38 ± 10.7 years, and the mean disease duration was 12.5 ± 7.3 years. The mean EDSS at DMF initiation was 3.0 ± 1.5 and the ARR in the previous year was 0.5. The mean BMI at baseline was 24.19 ± 4.48. Fifty patients were treatment-naïve (14.8%), while 257 switched to DMF from a first-line DMD, and 31 from second-line treatments. The latter 31 patients (2 with Mitoxantrone, 2 with Azathioprine, 21 patients with Fingolimod and 4 patients with Natalizumab), because of a possible overlap of immunosuppressive effects, observed a washout period sufficient to restore immune function and bring LC and WBC back within normal ranges. For patients switching from a first-line DMD, a wash-out period of at least 4 weeks was observed (longer if specifically reported by Summary of Product Characteristics or in accordance with treating-physicians' decisions).

**Table 1:** Baseline demographic, anthropometric, and clinical characteristics of the study population.

**Number of patients**	**338**
Sex: n (F/M)	197/141
Age at DMF beginning (years, mean± SD)	38 ± 10.7
Disease duration (years, mean± SD)	12.5 ± 7.3
EDSS at DMF initiation (mean± SD)	3.0 ± 1.5
Patients with comorbidities (%)	118 (34.9%)
BMI at DMF beginning (kg/m^2^, mean± SD)	24.19 ± 4.48
LC (cells/mm^3^ mean± SD)	1,940 ± 667.60
WBC (cells/mm^3^ mean± SD)	6380.8 ± 2051.93
**Previous DMDs**	
-Naïve: *n* (%)	50 (14.8%)
-Only first line treatments: *n* (%)	257 (76%)
-First and second line treatments; *n* (%)	31 (9.2%)

*DMF, Dimethyl fumarate; SD, standard deviation; EDSS, Expanded Disability Status Scale; BMI, Body Mass Index; LC, lymphocyte count; WBC, white blood cells; DMDs, Disease modifying drugs*.

### Temporal Profile of WBC and LC

**Figure 2:**
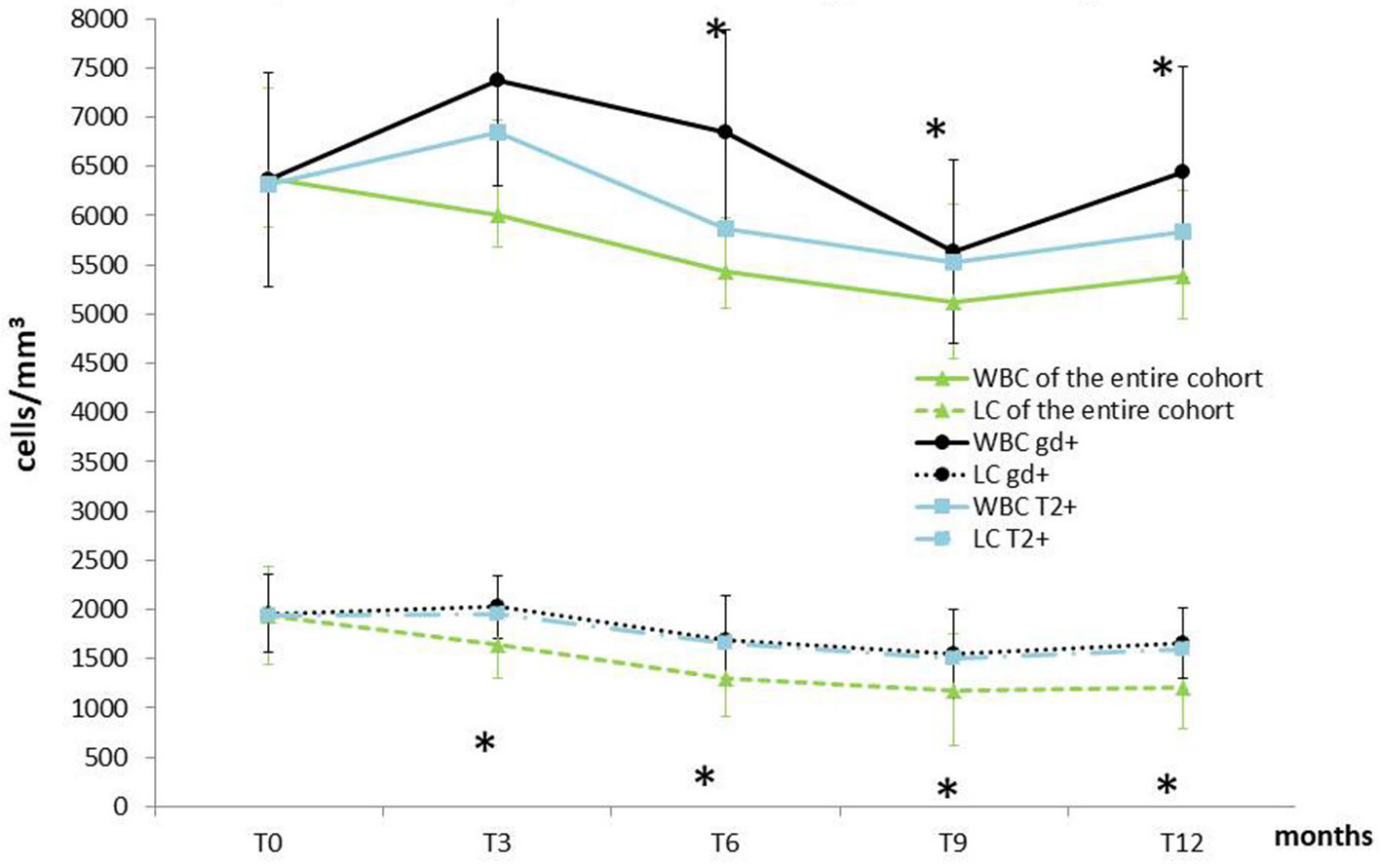
Temporal profile of WBC and LC of the entire cohort compared with patients showing MRI activity at T12. Data are presented as mean ± SD. WBC, white blood cell; LC, lymphocyte count; MRI, Magnetic Resonance Imaging; Gd+, Gadolinium enhancing; T2+, new hyperintense lesions; T0, baseline; T3, 3 months follow up; T6, 6 months follow-up; T9, 9 months follow up; T12, one year follow-up.^*^Indicates Wilcoxon matched pair test *p* < 0.05 (vs. T0) in the entire sample size.

Figure 2 shows WBC and LC values at the beginning of DMF and at 3-month intervals during the first year of treatment. We compared the LC and WBC between T0 and T3, and we observed a reduction of 15.4% in LC (from 1,940 ± 667.60 to 1642.18 ± 184.75; *p* < 0.0001) and a reduction of 5.9% in WBC (from 6380.80 ± 2051.93 to 6010.29 ± 1601.30; *p* = 0.124). In T0–T6, a reduction of 33.2% was observed in LC (from 1,940 ± 667.60 to 1296.06 ± 584.03; *p* < 0.001) and a reduction of 14.9% in WBC (from 6380.80 ± 2051.93 to 5,436 ± 1585.63; *p* < 0.001). In T0–T9, a reduction of 39.1% was observed in LC (from 1,940 ± 667.60 to 1181.86 ± 480.80; *p* < 0.001), and a reduction of 19.9% in WBC (from 6380.80 ± 2051.93 to 5119.28 ± 1252.66; *p* < 0.001). Finally, in T0–T12, a reduction of 37.14% was observed in LC (from 1,940 ± 667.60 to 1213.01 ± 577.6; *p* < 0.001) and a reduction of 15.7% in WBC (from 6380.80 ± 2051.93 to 5383.33 ± 1313.82; *p* < 0.001). From T3, 29 patients presented GRADE I CTCAE lymphopenia (< LLN-800 u/mm^3^), and 24 presented GRADE II lymphopenia (<800–500 u/mm^3^). The nadir of reduction was observed at T9, both for WBC (*p* < 0.0001) and LC (*p* < 0.0001), with a reduction, respectively, of 19.8 and 39.1% from T0.

### Correlation Between WBC and LC Variation and Disease Activity

During the first year of treatment, in our cohort, the ARR was significantly lower than ARR as recorded in our clinical database for the previous year (0.25 vs. 0.51, *p* = 0.001). We evaluated the correlation between WBC and LC variation and outcomes of disease activity during DMF-treatment (Table 2).

**Table 2:** LC and WBC of the patients stratified for Gd+ lesions, new T2 lesions and relapses.

	**GD+ lesions at baseline MRI (YES) *N* = 95 (28%)**	**GD+ lesions at baseline MRI (NO) *N* = 243 (72%)**	**Gd+ lesions at T6 (YES) *N* = 48 (14.2%)**	**Gd+ lesions at T6 (NO) *N* = 290 (85.8%)**	**Gd+ lesions at T12 (YES) *N* = 30 (8.9%)**	**Gd+ lesions at T12 (NO) *N* = 308 (91.1%)**
Baseline LC (cells/mm^3^)	1,846 ± 554	1,986 ± 720	1,857 ± 584.4	2,102 ± 912.4	1,903 ± 503	1,967 ± 498
Baseline WBC (cells/mm^3^)	6,300 ± 1,636	6,466 ± 2,222	6,630 ± 2,033	6,228 ± 1,995	6,630 ± 917	6,204 ± 1,649
LC at T3 (cells/mm^3^)	1,925 ± 1,281	1,588 ± 583	1,845 ± 455^*^	1,486 ± 556^*^	2,024 ± 337	1,518 ± 581
WBC at T3 (cells/mm^3^)	6,208 ± 1,326	6,006 ± 1680	5,875 ± 1,277	5,788 ± 1624	7,376 ± 1165	5,962 ± 1,575
	**New T2 lesions at baseline MRI (YES)** ***N*** **=** **125 (37%)**	**New T2 lesions at baseline MRI (NO)** ***N*** **=** **213 (63%)**	**New T2 lesions at T6 (YES)** ***N*** **=** **91(26.9%)**	**New T2 lesions at T6 (NO)** ***N*** **=** **247 (73.1%)**	**New T2 lesions at T12 (YES)** ***N*** **=** **50 (14.8%)**	**New T2 lesions at T12 (NO)** ***N*** **=** **288 (85.2%)**
Baseline LC (cells/mm^3^)	2,069 ± 740	1,880 ± 642	1,855 ± 546.1	2021.63 ± 837.5	1903.8 ± 614	1,941 ± 393
Baseline WBC (cells/mm^3^)	6,344 ± 2,244	6,478 ± 2,000	6,001 ± 2,024	6,419 ± 1,986	6,312 ± 1,090	6,234 ± 1,678
LC at T3 (cells/mm^3^)	1,811 ± 1,826	1,518 ± 570	1,739 ± 571	1,461 ± 653	1,952 ± 317^*^	1,495 ± 592^*^
WBC at T3 (cells/mm^3^)	6,247 ± 1,539	5,936 ± 1,357	5,969 ± 1,392	5,729 ± 1,641	6,840 ± 1,073	5,963 ± 1,940
	**Relapse before DMF (YES)** ***N*** **=** **152 (45%)**	**Relapse before DMF (NO)** ***N*** **=** **186 (55%)**	**Relapse within T6 (YES)** ***N*** **=** **41 (12%)**	**Relapse within T6 (NO)** ***N*** **=** **297 (88%)**	**Relapse within T12 (YES)** ***N*** **=** **63 (18.6%)**	**Relapse within T12 (NO)** ***N*** **=** **275 (81.4%)**
Baseline LC (cells/mm^3^)	1,837 ± 622	2,027 ± 696	2054.18 ± 956	1,858.15 ± 561	1,702 ± 549	1,868 ± 858
Baseline WBC (cells/mm^3^)	6,194 ± 2,073	6,553 ± 2,033	6,324 ± 2,126	6,216 ± 1,972	6,165 ± 1,045	5,600 ± 1,275
LC at T3 (cells/mm^3^)	1,551 ± 574	1,725 ± 1,548	1,687 ± 353	1,597 ± 537	1,761 ± 465^*^	1,386 ± 402^*^
WBC at T3 (cells/mm^3^)	6,193 ± 1,662	5,841 ± 1,537	6,243 ± 1,398	5,850 ± 1,688	6,031 ± 1,687^*^	5,660 ± 1,110^*^

*Values expressed as mean (±SD)*.

*LC, lymphocyte count; WBC, white blood cells; T3, 3 months follow-up; T6, 6-months follow up; T12, 1 year follow up*.

* *underlines p <0.05*.

In the univariate analysis, patients who experienced relapses within the first year of treatment had a higher WBC count at T3 (6,031 ± 1,687/μl vs. 5,660 ± 1,110/μl, *p* = 0.05) as well as a lower variation in WBC between T0 and T3 (−59.53 ± 1006.33 vs. 167.79 ± 1660.78, *p* = 0.05) than patients who experienced no relapse (Table 2). However, multivariate analysis confirmed the reduction in WBC as a predictive factor for relapses occurring during the entire first year of treatment (OR = 1.054, 95% CI = 1.03–2.65; *p* = 0.043, Table 3).

**Table 3:** Predictors of relapses and MRI disease outcomes at T12: multivariate logistic regression analysis.

	**OR (95% CI); p-value**		
**Variable**	**Relapse within T12**	**New T2 lesions at T12**	**Gd+ lesions at T12**
Gender (F)	0.635 (0.09–4.13); 0.630	0.313 (0.06–15.14); 0.557	0.095 (0.001–5.23); 0.566
Age	0.851 (0.697–1.04); 0.060	0.63 (0.56–11.25); 0.991	0.411 (0.062–2.73); 0.358
Baseline Gd+ (YES)	0.045 (0.005–0.44); 0.080	0.032 (0.001–1.23); 0.994	0.011 (0.001–1.96); 0.088
Baseline T2 (YES)	1.99 (0.25–16.07); 0.510	1.22 (0.28–2.21); 0.198	0.31 (0.074–1.29); 0.109
Relapses pre-DMF (YES)	0.73 (0.61-1.60); 0.060	1.016 (0.175–14.5); 0.993	1.76 (0.175–15.32); 0.998
1^st^ line DMD vs. naive	1.002 (0.045–3.47); 0.990	6.37 (0.19–7.52); 0.988	0.15 (0.001–69.9); 0.545
2^nd^ line DMD vs. naive	0.236 (0.008–6.74); 0.380	2.25 (0.03–2.29); 0.862	0.31 (0.07–1.3); 0.109
Variation in WBC	1.054 (1.03-2.65); 0.043	–	–
LC at T3	1.08 (0.98–2.80); 0.090	1.01 (1.00–1.95); 0.005	1.003 (1.00–1.07); 0.046
Baseline BMI	0.828 (0.61–1.15); 0.213	0.99 (0.98–1.00); 0.026	0.71 (0.52–0.98); 0.037
DIHL at T3	2.5 (0.77–8.05); 0.125	1.26(1.07–1.96); 0.040	1.31(1.12–5.34); 0.043
DIHL at T6	0.62 (0.16–2.42); 0.493	1.48 (1.15–2.59); 0.035	1.14 (1.133–6.33); 0.050

WBC, white blood cells; BMI, Body Mass Index; LC, lymphocyte count; DIHL, high number of DMF-induced lymphocytes; T3, 3 months follow-up; T6, 6-months follow up; T12, 1 year follow up. ^*^Models always considered as covariates baseline clinical and demographic characteristics of the cohort, and those covariates significant at the univariate analysis. Underlined values are those statistically significant (p<0.05).

We observed no differences in the absolute value or the delta of WBC between patients presenting new Gd+/T2 lesions and patients who did not. On the other hand, LC was higher at T3 in patients who experienced new Gd+ lesions at T6 (1,845 ± 455 vs. 1,486 ± 556, *p* = 0.043), in patients who experienced new T2 lesions at T12 (1,952 ± 317 vs. 1,495 ± 592; *p* = 0.040), and in patients experiencing a relapse during the first year of treatment (1,761 ± 465 vs. 1,386 ± 402; *p* = 0.033) (Table 2). Multivariate analysis showed that LC at T3 was a predictive factor for Gd+ lesions at T12 (OR = 1.003, 95% CI = 1.00–1.07; *p* = 0.046) (Table 3) and new T2 lesions at T12 (OR = 1.01; 95% CI = 1.00–1.95; *p* = 0.005) (Table 3).

In order to investigate the impact of the lymphocytes on the risk of relapses and on worsening MRI, the median values of LC at T3, T6 and T9 were calculated, and the population was divided into two groups:

- Patients with LC below the median calculated (with low number of DMF-induced lymphocytes -DILL-),
- Patients with LC above the median calculated (with high number of DMF-induced lymphocytes -DIHL-).

In the univariate analysis, during the first and second semesters of treatment, no differences were found between the clinical activity of patients with DIHL and patients with DILL (Table 2). However, considering MRI activity at T12, patients with DIHL at T3 (OR = 1.31, 95% CI = 1.12–5.34, *p* = 0.043) and at T6 (OR = 1.14, 95% CI = 1.13–6.33; *p* = 0.05) had a higher risk of Gd+ lesions (Table 3). Patients with DIHL at T3 (OR = 1.26, 95% CI = 1.07–1.96; *p* = 0.04) and at T6 (OR = 1.48, 95% CI = 1.15–2.59; *p* = 0.035) also had a higher risk of new T2 lesions at T12, compared to patients with DILL (Table 3).

### Role of BMI

**Figure 3:**
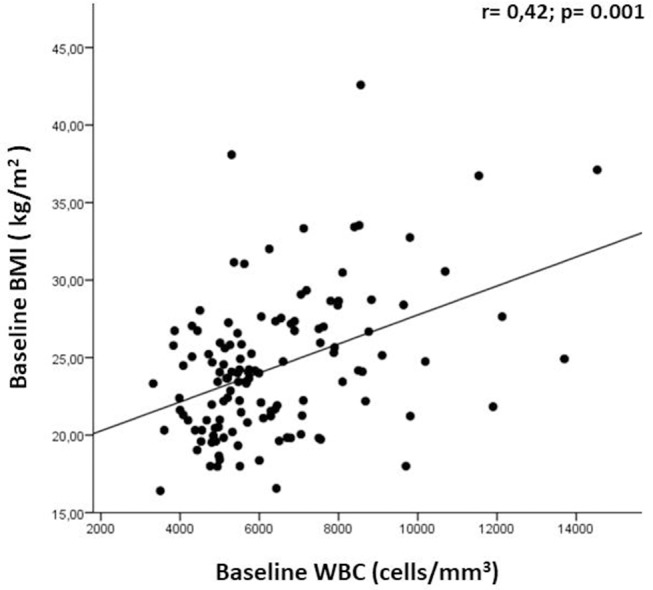
Correlation between baseline BMI and WBC. WBC, white blood cell; BMI, Body Mass Index.

Patients experiencing a relapse during the first year of treatment presented a lower baseline BMI (20.9 ± 2.4 vs. 24.7 ± 4.4; *p* = 0.001) (Table 2). Multivariate analysis, confirmed baseline BMI as a predictive factor for GD+ lesions at T12 (OR = 0.71; CI = 0.52–0.98; *p* = 0.037) (Table 3) and also for new T2 lesions at T12 (OR = 0.99, 95% CI = 0.98–1.00; *p* = 0.026) (Table 3). Figure 3 graphically represents the BMI in patients with and without new T2 lesions at T12.

**Figure 4:**
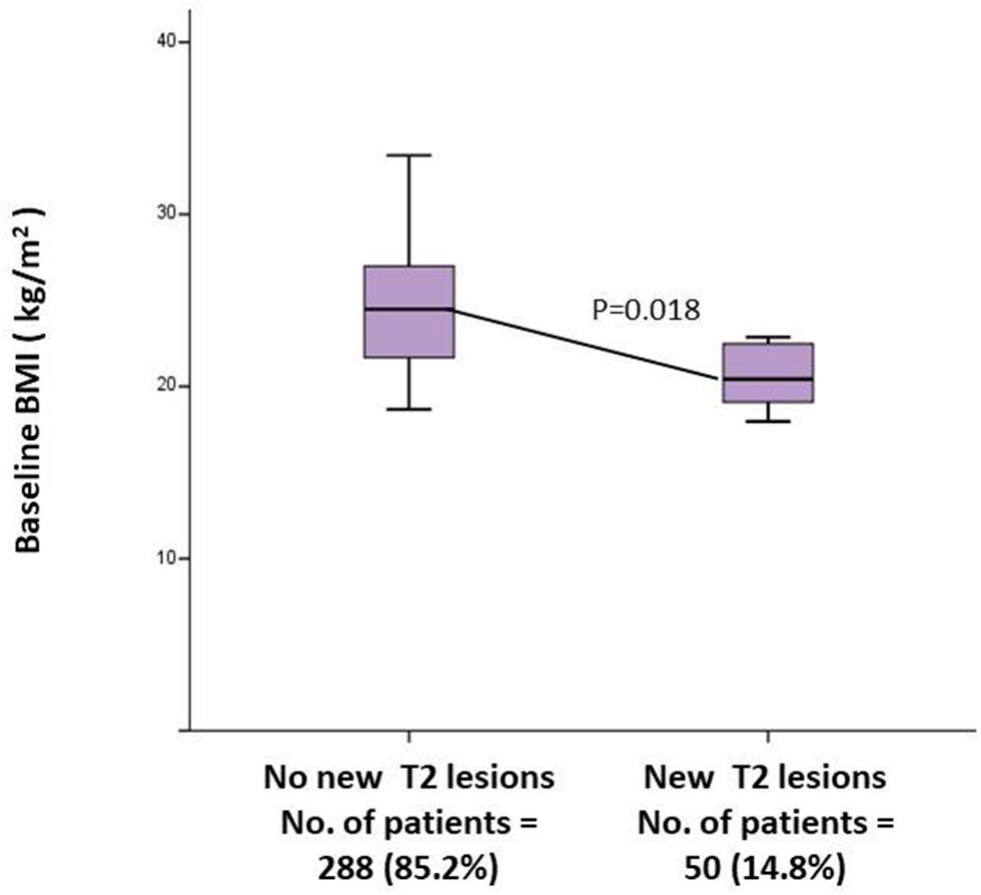
Univariate between baseline BMI and MRI activity at T12. BMI, Body Mass Index; MRI, Magnetic Resonance Imaging; T12, 1 Year follow up.

At T0 we observed a direct correlation between the WBC count and BMI (*r* = 0.42; *p* = 0.001; Figure 4); we had also expected a correlation between BMI and LC, as often found in other diseases, but we did not observe this. No other correlations were found between baseline WBC and LC values and any clinical-demographic variable (baseline age and gender, disease duration, number of relapses within the previous year, previous DMDs, presence/absence of new/enlarging T2 lesions, and presence/absence of Gd+ lesions). We further investigated whether any comorbidities, presented by 34.9% of our population (118 patients), could influence BMI at baseline, but we found no difference between BMI in patients with and without comorbidities (24.7 ± 3.8 vs. 23.9 ± 5.4; *p* = 0.117). We also observed a direct correlation between BMI and WBC at T3 (*r* = 0.23; *p* = 0.016); no other correlations were found between baseline BMI and variations in WBC and LC values.

## Discussions and Conclusions

Several DMDs used to treat MS can reduce the occurrence of relapses and slow the progression of neurological disability, thus improving patients' quality of life. However, variability in response to DMDs in patients with MS represents a significant clinical challenge. Potentially, treatment delays may occur in suboptimal responder patients, exposing them to adverse effects without significant benefit. Therefore, biomarkers of treatment response are urgently needed. In our cohort, after 1 year of therapy, DMF was effective in reducing both clinical and MRI disease activity, as shown by the multivariate models used that take into account the absence of exact date of both clinical and MRI relapses. We tried to identify some possible factors that could influence DMF effectiveness, avoiding potential confounders such as baseline disease activity and other patient specific characteristics that were considered in our analysis. Building our hypothesis we also considered the drug effects that involve immunomodulatory action as well as neuroprotection (10, 18). DMF, in fact, reduces cytokine production (19), down-regulates the migratory activity of immune cells at the blood–brain barrier (20) and activates the antioxidant nuclear factor NRF2 transcriptional pathway (10, 21). Considering that the Nrf2 pathway plays an important role in energy metabolism (11, 22) and that recent data demonstrate that DMF is a negative regulator of preadipocyte differentiation mediated by STAT3 inhibition (23), we tried to link patients' BMI with both clinical (relapses) and MRI outcomes. There have been studies indicating that overweight and obese patients have a lesser chance of obtaining complete remission and Non-Evidence of disease Activity (NEDA)-status during IFN-β-treatment, although it remains to be determined whether this is due to a suboptimal treatment response or to a generally more aggressive disease (24). Moreover, Krupp et al. have argued that a higher BMI in US adolescents could be a relevant explanation for their worse outcomes during IFN-ß therapy (25). Obesity, however, is known to effect serum inflammatory markers, promoting a chronic low-grade inflammatory state (26), also through the activation of the nuclear factor (NF) κB pathway (27), and DMF has been shown to prevent the induction of NFκB dependent transcription (28). In our cohort, a lower BMI was a predictive factor for MRI activity at T12, and we tried to explain this finding with the differences in DMF action according to the amount of adipose tissue; we may hypothesize that the higher the amount of adipocytes, the greater the action that DMF can exert on the inflammatory aspect of the disease. Nowadays, in fact, adipocytes are attributed a metabolically active role in biochemical mechanisms that may contribute to a chronic low-grade inflammatory status (29), increasing ROS oxidative stress. Reactive oxygen and nitrogen species have been described to induce damage to biological macromolecules in MS lesions (30, 31). Moreover, in RR and also in progressive MS, active lesions are associated with inflammation (32), although not always present in the latter form of the disease.

We also found that LC at T3 was a predictive factor for clinical and MRI activity. DMF suppresses lymphocytes and induces T-cell apoptosis. DMF-induced lymphopenia could theoretically be linked to disease control. However, in DEFINE and CONFIRM, the reduction in ARR at 2 years in patients treated with DMF 240 mg bid vs. placebo was not substantially different in patients with lymphopenia (≥1LC < lower limit of normal) compared to those without lymphopenia (all LCs ≥lower limit of normal) (7). However, real-life data are sometimes discordant on this point; for example, data from a Dallas Multiple Sclerosis Center (33) have shown a greater risk of relapses in patients with higher LC at 3 months [*p* < 0.001, hazard ratio [HR]: 1.82], just as we found in our cohort. Moreover, the Dallas examiners stratified LC by tertile, and found a reduced risk in patients with lower LC values: 1,200 cells/mL compared with mid-tier (1,210–1,800 cells/mL) and the highest tertile (>1,810 cells/mL) (*p* < 0.01). We obtained comparable results by stratifying patients according to the LC median during the follow-up, and found that patients with DIHL at T3, T6, and T12 had a higher risk of MRI activity than those with DILL. Conversely, in another cohort from the MS Center of Washington (34), DMF-induced lymphopenia did not predict a good clinical response to therapy. In this Washington study, the authors examined predictive factors of lymphopenia including recent natalizumab exposure as risk factors of developing moderate to severe lymphopenia during treatment with DMF. In our cohort, the previous treatment was not associated with a different incidence in reduction of WBC or LC, and we found no significant correlation between the previous DMD and the disease activity. However, we observed a direct correlation between the WBC count and BMI (*r* = 0.32; *p* = 0.001) at T0; since fat tissue releases inflammatory cytokines, this may explain why in our cohort we found a direct correlation between WBC (which can be considered a non-specific marker of inflammation) and BMI. Moreover, while BMI and LC are independently linked to DMF effectiveness, the association test performed on these did not prove to be a statistically significant effect of either clinical or MRI activity. The reason for this may be that our cohort contained few underweight or obese patients. Another possible explanation could be that the two variables did not change to the same extent: BMI remained constant during the observation period, while there was a reduction (15.36%) in LC which was statistically significant from the third month and tended to stabilize around the ninth month. This finding differs from what has been observed in an integrated analysis of long-term extension studies on DMF (7), which demonstrated that LC decreased by 30% during the first year and then plateaued, remaining above the lower limit of normal (LLN).

Our analysis did not investigate the influence of different lymphocytes subsets (LS) on disease activity during DMF. A recent study demonstrated that patients under DMF therapy who remained stable (with no radiological or clinical evidence of disease activity) tended to exhibit greater reductions of CD3+, CD4+, CD8+, and CD19+ cells compared to active MS patients, and they also presented significantly higher CD4/CD8 ratios (35). However, since the monitoring of the immune system re-modulation in DMF relapsing MS patients is not required during the routine clinical practice, we did not perform, in this real life setting, analyses on specific LS variations and this is one of the limitations of our study. Other limitations may be the lack of a control group treated with a different DMD, as well as the lack of details regarding metabolic parameters of patients (blood glucose levels and urine tests), the metabolic state of immune cells, and patients' consumption behavior. Further studies will be performed that will include these details in order to have a more in-depth analysis of the role of BMI in predicting DMF effectiveness. Another limitation is that, given the oral administration of the drug, we did not check patients' adherence. Finally, the most important limitation may be the observational design of our study: our findings may only reflect a unique sample population and, thus, may not be generalized to other groups.

In conclusion, elucidating the significance of DMF-induced lymphopenia could be important for clinical decisions, including the frequency of monitoring and the possibility of predicting both clinical or MRI activity. This becomes an even more appealing prospect when it is considered that changes in LC can be detected from the third month of treatment, and might therefore offer the possibility of formulating an early prediction of a possible suboptimal response. Moreover, the use of an easily detectable baseline characteristic (BMI), could allow better profiling of patients for a tailored treatment. However, given the observational nature of our work, there is a need for further studies on larger cohorts in order to support the clinical importance of our results.

## Ethics Statement

This study was carried out in accordance with the recommendations of name of guidelines, name of committee with written informed consent from all subjects. All subjects gave written informed consent in accordance with the Declaration of Helsinki. The protocol was approved by the Ethical Committee of the University of Bari.

## Author Contributions

AM participated in the design of the study, collected the data, and drafted the manuscript. AI collected the data and revised the manuscript, helped to give final approval. GL participated in the design of study, performed statistical analysis and helped to given final approval. MD participated in the design of study and revised the manuscript. DM revised the manuscript and helped to give final approval. VF revised the manuscript. PI helped to draft the manuscript. MT revised the manuscript and helped to give final approval. DP conceived of the study, and participated in its design, revised the manuscript and helped to give final approval of the version to be published.

### Conflict of Interest Statement

DP received honoraria for consultancy and/or speaking from Biogen Idec, Merck-Serono, Almirall, Sanofi-Aventis, TEVA, Novartis and Genzyme. PI has served on scientific advisory boards for Biogen Idec and Bayer, and has received funding for travel and/or speaker honoraria from Genzyme, Sanofi-Aventis, Biogen Idec, Teva and Novartis. MT received honoraria for consultancy or speaking from Biogen, Sanofi Aventis, Merck Serono, Novartis, Genzyme, TEVA, and Bayer-Schering and research grants from Merck Serono, Biogen, and Novartis. The remaining authors declare that the research was conducted in the absence of any commercial or financial relationships that could be construed as a potential conflict of interest.
